# Semi-continuous anaerobic digestion for biogas production: influence of ammonium acetate supplement and structure of the microbial community

**DOI:** 10.1186/s13068-015-0197-z

**Published:** 2015-02-05

**Authors:** Haijia Su, Luo Liu, Shaojie Wang, Qingfeng Wang, Yixin Jiang, Xiaocong Hou, Tianwei Tan

**Affiliations:** Beijing Key Laboratory of Bioprocess, Beijing University of Chemical Technology, No.15, Beisanhuan East Road, Beijing, 100029 People’s Republic of China

**Keywords:** Anaerobic digestion, Biogas production, Ammonium acetate, Glucose, Microbial community

## Abstract

**Background:**

As an efficient disposal method of food waste, anaerobic digestion (AD) for biogas production is widely used. In order to understand the enhanced efficiency and stability of AD by appropriate amounts of ammonia and volatile fatty acids (NH_4_^+^/VFAs), the characteristics of the corresponding microbial community with ammonium acetate supplement were investigated by denatured gradient gel electrophoresis (DGGE) and pyrosequencing analyses of samples, with or without supplement of NH_4_^+^/VFAs.

**Results:**

In this study, four different supplement strategies of adding ammonium acetate were investigated, including a blank group (without supplement of ammonium acetate), a low group (L group, 0.7 g/L/d), a moderate group (M group, 1.0 g/L/d) and a high group (H group, 1.3 g/L/d), respectively. The average daily gas production was 1,839 mL/d, 1,655 mL/d, 1,448 mL/d and 1,488 mL/d for L, M, H and blank groups, respectively. The results reveal that the absence or overload of NH_4_^+^/VFAs leads to the inhibition or failure of the AD operation. The blank and H groups were selected for further investigation of the microbial community by DGGE and pyrosequencing analyses. A significant difference of the microbial communities at different AD stages was observed between the blank and H groups.

**Conclusions:**

Ammonium acetate, as an efficient supplement, significantly influences the characteristics of a semi-continuous AD operation. The DGGE and pyrosequencing analyses indicated that the different bacterial and archaeal communities occurred in the blank and H groups at different AD stages. Thus, an appropriate ammonium acetate supplement may maintain the balance of the microbial community and could be applied to adjust the AD operation and microbial composition towards optimal biogas production.

## Background

As an efficient disposal method in the treatment of food, and fruit or vegetable wastes [1-3], anaerobic digestion (AD) for biogas production is widely used. As previous research has reported [4], the AD process includes steps of hydrolysis, acidogenesis, acetogenesis and methanogenesis. In the first three steps, the bacterial community at phylum level is commonly dominated by *Proteobacteria*, *Firmicutes* and *Bacteroidetes* [5-7], which generate sufficient volatile fatty acids (VFAs) for the methane production. The archaeal community is, however, often dominated by the *Euryarchaeota phylum*, which utilizes acetate, H_2_, CO_2_ and methyl compounds for methane production. The stability of AD operation was affected significantly by the microbial communities, whose diversity was directly affected by varying digestion conditions [8].

The operation mode of AD and its parameters [9], the pretreatment of the substrate [10] and the added C- and N-resources [11-13] were studied in view of AD operational stability. Whereas the addition of metal-ions could maintain a stable and efficient digestion [12], the effect of adding other additives, especially ammonia (NH_4_^+^), has not been investigated in detail, although an overload of ammonia can hamper the aerogenesis in AD and reduce the activity of methano-bacteria [14,15]. Some previous research has indicated that NH_4_^+^/VFAs could form a weak buffer system at the beginning of the digestion operation, thus enhancing the tolerance of AD, whilst the failure of the buffer action resulted in a direct inhibition [16].

Wang *et al*. reported that the formed buffer of NH_4_^+^/VFAs during the semi-continuous AD process boosted the biogas production and efficiently maintained the AD stability in the treatment of food waste. When ammonia acetate was added to the AD, the liquid environment changed and caused a shift of microbial communities [17]. Previous studies focused upon the AD microbial community analysis, either during the domestication or the inhibition period [18-20].

The relationship of the exogenous buffer impact and the microbial communities was not previously investigated; thus it is the objective of the present work to investigate the effect of different ammonium acetate concentrations on the AD characteristics and the change in microbial communities. In order to eliminate the complex influence of other factors, a solution of glucose with ammonia acetate was used to simulate the food waste.

The key objectives of this work involve: (i) studying the effects of different ammonium acetate supplement strategies on the biogas and CH_4_ production, and on the liquid characteristics during the AD process, whilst evaluating the stability and inhibition of AD when exogenous buffer is added; and (ii) assessing the effect of the supplement onto the microbial communities to identify the microbial communities’ change under different operating modes.

## Results and discussion

### Effect of different ammonium acetate concentrations on the characteristics of anaerobic digestion

Biogas production trends for the blank and supplement groups with different addition strategies (low (L), moderate (M) or high (H)) were assessed. The average daily gas production was 1,839 mL/d, 1,655 mL/d, 1,448 mL/d and 1,488 mL/d for L, M, H and blank groups, respectively. The H and blank groups were gently inhibited after about 25 days, with average daily biogas production of 1,266.9 mL/d and 1,256.8 mL/d at 51 days as listed in Table 1, respectively. The ammonium acetate supplement moreover caused an accelerated startup in the groups with ammonium acetate supplement. Due to the VFAs inhibition, the daily average biogas production of the blank group during approximately 26 to 51 days decreased by 15% in comparison with the period 0 to 25 days (approximately). The results clearly stress the importance of adding a stable N-resource supplement as the key factor to the stability and high efficiency of the AD system.

**Table 1:** **Effect of ammonium acetate concentration on characteristics of anaerobic digestion**

**Group**	**Different period (days)**	**Average daily total biogas production (mL)**	**Average daily CH**_**4**_ **concentration (%)**	**NH**_**4**_^**+**^ **(g/L)**	**Volatile fatty acids (g/L)**
L	10	1,837.0 ± 46.4	61.66 ± 1.56	1.47	2.44
	25	1,767.5 ± 48.3	61.14 ± 2.38	1.86	4.88
	51	1,880.4 ± 37.9	59.11 ± 1.62	1.97	8.00
M	10	1,757.3 ± 54.1	63.38 ± 1.52	1.27	2.83
	25	1,708.5 ± 37.1	61.80 ± 2.17	2.73	5.69
	51	1,586.4 ± 40.8	54.53 ± 2.08	3.22	8.70
H	10	1,710.5 ± 56.3	64.39 ± 1.81	1.48	3.80
	25	1,586.5 ± 43.6	61.39 ± 1.69	3.35	9.69
	51	1,266.9 ± 49.6	45.86 ± 1.96	5.97	15.44
Blank	10	1,678.0 ± 42.4	52.42 ± 1.66	0.15	3.63
	25	1,778.3 ± 53.1	56.65 ± 2.20	0.12	6.44
	51	1,256.8 ± 51.6	47.08 ± 2.12	0.27	9.55

The groups with the supplement maintained a significantly higher methane production during the whole process. The methane production of L, M and H groups was 44.5%, 26.5% and 4.1% higher than that of the blank group, respectively. However, after 25 days, the NH_4_^+^ inhibition clearly occurred in the H group. The average methane production of the H group at the end of the AD operation (about 51 days) was only 581.0 mL/d; 96.3% and 57.0% lower than that of L and M groups, respectively. Similarly, the VFAs inhibition of the blank group also limited the methanogen activity during the end period of the digestion (approximately 26 to 51 days) (see Table 1).

Whereas the VFAs inhibition occurred in the blank group, a weaker buffer in the L and M groups was formed from the ammonium acetate as supplement, thus defusing the impact of the VFAs inhibition and resulting in a higher methane production. However, an overload of ammonium acetate also caused NH_4_^+^-inhibition directly, which was more obvious in later periods of the M and H groups, especially in the H group.

**Figure 1:**
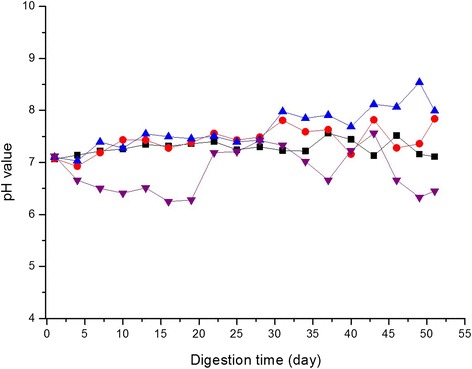
**Effect of different ammonium acetate concentrations on the pH value of the anaerobic digestion (AD).** The different amount of ammonium acetate was supplemented at L group (black square), M group (red circle symbol), H group (blue triangle symbol) and blank sample (violet triangle symbol). The experiments were operated in a 1 L fermentor at a mesophilic temperature of 37 ± 1°C for the semi-continuous AD.

As shown in Figure 1, the supplement of ammonium acetate affected the pH value differently. An instable pH change was obvious in the blank group, without the N-resource supplement; below 7.0 during the whole process. However, the pH value of L and M groups were relatively stable during the whole process, because a weaker buffer system of NH_4_^+^/VFAs was formed due to the supplement of ammonium acetate, which weakened the VFAs impact. Nevertheless, the pH of the H group showed a severe increase to 8.0 after about 25 to 30 days, which can be attributed to a continuous NH_4_^+^ accumulation, followed by NH_4_^+^ inhibition.

The effect of different supplement strategies on the NH_4_^+^ concentration from different stages was shown in Table 1, where the blank group maintained the lowest NH_4_^+^ concentration during the whole process; the highest NH_4_^+^ concentration in the blank group was only 0.27 g/L during the whole AD process. With a continuous VFAs accumulation, VFAs inhibition resulted in a poor biogas and methane performance of the blank group during the whole process (see Table 1).

The fastest NH_4_^+^ accumulation rate was obtained in the H group, but the AD operation would be hampered at NH_4_^+^ concentration in excess of 3 g/L after a period of about 20 to 25 days. The average biogas production decreased from 1,586.5 to 1,266.9 mL/d and the average methane concentration decreased from 61.4 to 45.9%, comparing day 25 with 51, respectively. The present study indicates that the critical point of NH_4_^+^ concentration was about 2.5 to 3.0 g/L, so it is remarkable that the M group was also slightly inhibited when the NH_4_^+^ concentration reached 2.7 g/L, at around 25 days. However there was no obvious difference in the biogas and methane production of the L group during the whole AD process, maintaining the optimum productive capacity.

Table 1 also clearly shows the variation of VFAs accumulation in the different groups during the whole AD process. The VFAs concentration in the blank group increased significantly from 3.6 to 9.6 g/L. Similarly, Table 1 illustrates that the VFAs concentration in the L and M groups were slightly lower than that of the blank group, but these two groups maintained a stable biogas production without inhibition. However, the higher ammonium acetate supplement caused a severe VFAs increase in the H group, reaching 15.4 g/L at the end.

A weak buffer system between VFAs and NH_4_^+^ in the AD operation was necessary to maintain its high efficiency and stability. Both absence and overload of NH_4_^+^/VFAs will directly lead to the inhibition or failure of the AD operation.

### Denatured gradient gel electrophoresis analysis of bacterial and archaeal communities at different anaerobic digestion stages

**Figure 2:**
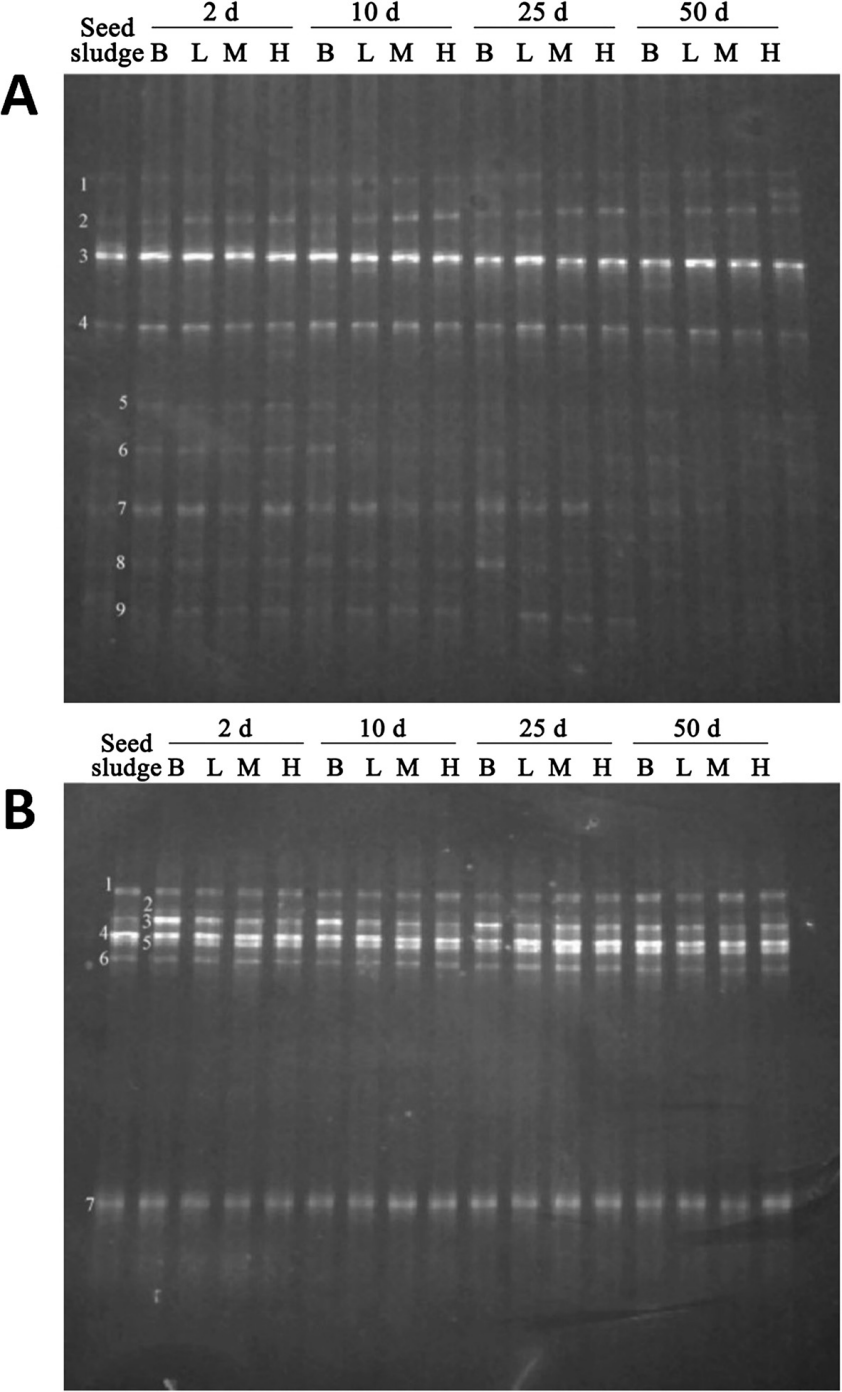
**The DGGE profile for the microbial communities analyses at different AD stages. A**, Bacterial community at different AD stages; **B**, Archaeal community at different AD stages. AD, anaerobic digestion; B, blank group; D, days; DGGE, denatured gradient gel electrophoresis; H, high group; L, low group; M, moderate group. The bands in Figure 2 **A** indicate the presence of different microorganisms: 1.*Thermotogae* bacterium JX473505.1; 2.Uncultured bacterium DQ490120.1; 3.Uncultured *Planctomycetales* bacterium AM406812.1; 4.*Mesotoga* sp.KC800693.1; 5.*Thermotogae* bacterium JX473505.1; 6.*Trichococcus* sp.FJ374769.1; 7.Uncultured *Spirochaetes* bacterium JN998192.1; 8.*Trichococcus* sp.AM933652.1; 9.*Synergistetes* bacterium JX473564.1.The bands in Figure 2 **B** indicate the presence of different microorganisms: 1.*Methanobacterium formicicum* JX042445.1; 2. *Methanobacteriaceae* archaeon JN836400.1; 3.Uncultured *Methanobacterium* sp.HQ231787.1; 4.Uncultured methanogenic archaeon DQ682552.1; 5.Uncultured *crenarchaeote* HQ141818.1; 6.Uncultured methanogenic archaeon DQ682560.1; 7.Unkonw.

Throughout the AD, adding exogenous buffer would generate a significant effect on the microbial communities, as shown in the denatured gradient gel electrophoresis (DGGE) results. The microbial structure of the community in each group throughout the AD process changed at different stages, but the dominant bacteria did not change too much, as shown in Figure 2. With the extension of the digestive time, some bacteria in the blank and H groups were inhibited, as reflected to the DGGE bands, reducing in brightness or even disappearing.

A bacterial community change was also observed from Figure 2A. At the beginning stage of AD, the DGGE bands were all similar, with a slight change due to the seed sludge. A difference of the DGGE bands between different groups was observed after 10 days, and the difference increased with the progress of AD. Regarding the biogas production, before 25 days all groups exhibited a stable biogas production, while the biogas production began to decrease both in the blank and H groups in the last stage (Table 1). This indicated that the initial microbial communities had the capability of adaptation and self-regulation, with a slight effect on the overall biogas production. However, after a certain change in microbial communities, a reduced biogas production was observed. It is likely that in this case the ability of the bacterial community to self-repair may be insufficient.

**Figure 3:**
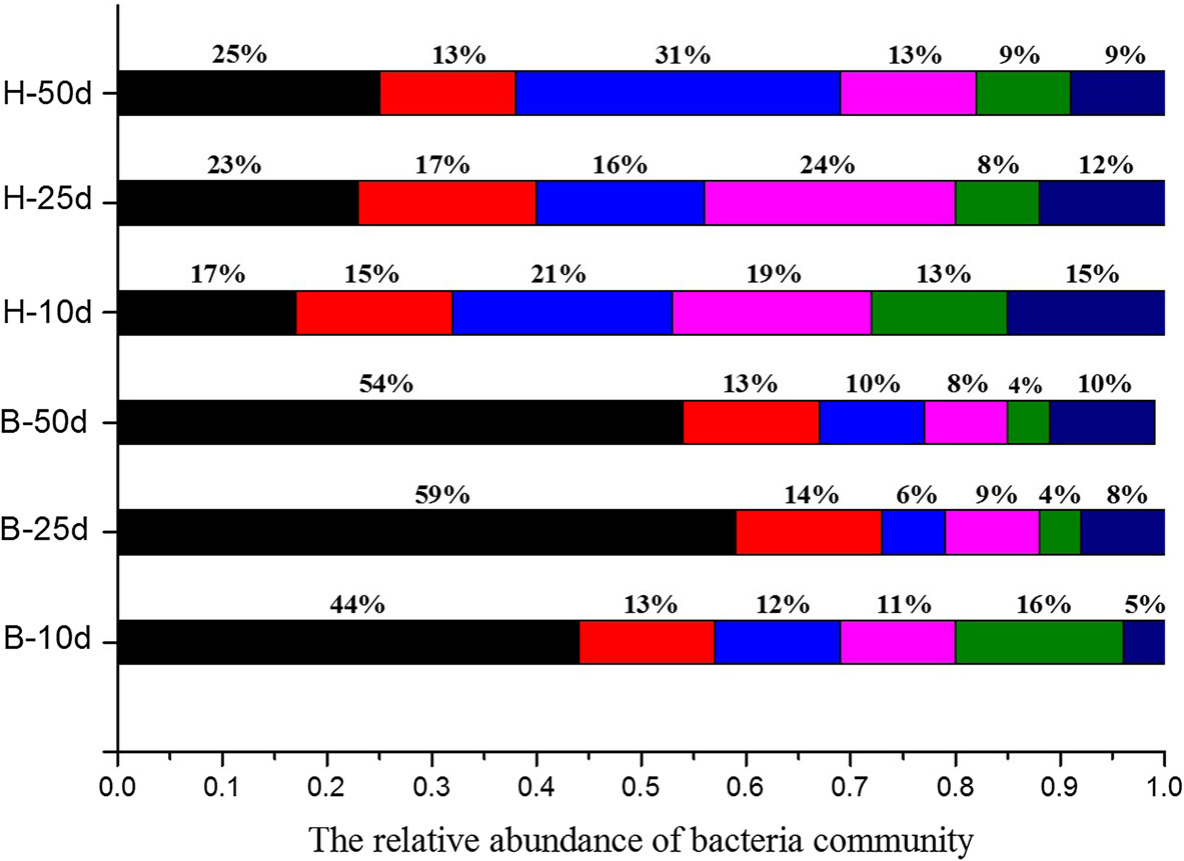
**Taxonomic composition of the bacterial community at phyla level in each sample.** The relative taxonomic abundances of the bacteria community at the phylum level. Phyla *Firmicutes* (black rectangle), WWE1 (green rectangle symbol), *Bacteroidetes* (red rectangle symbol), *Proteobacteria* (blue rectangle symbol), *Spirochetes* (pink rectangle symbol) and other (dark blue rectangle symbol) dominated in both blank and H groups.

For further understanding of the effect of adding an exogenous buffer system on the bacterial community between the groups showing a significant difference, we selected samples of the blank and H groups at 10, 25 and 50 days for pyrosequencing analysis in depth (Figure 3).

The DGGE analysis was carried out for the archaeal community as well. No significant change was observed throughout the entire AD for all groups (Figure 2B). In order to study the effect of ammonia acetate on the archaeal community, we choose the H group as the research object since it had the highest ammonia concentration. Further analysis of the archaeal *16S rRNA* gene by pyrosequencing was shown in the follow text.

### Bacterial ***16S rRNA*** variation under different inhibitory conditions

The above research showed that certain operation factors could cause inhibition effects in the AD process, with effect on the biogas production, but could also be expected to influence the diversity of the microbial communities. In order to correlate the ammonium acetate supplement and AD microbial communities, especially under the inhibitory conditions, the microbial communities of the blank and H groups were selected for further investigation by pyrosequencing analysis.

Discarding the unquantified sequences, a total of 23,703 effective sequences were obtained. Effective sequences of 2,700 were randomly picked from each sample which guaranteed the same analyzed sequencing depth. At the species level, observed numbers of operational taxonomic units (OTUs) were quite different from the estimated (abundance-based coverage estimator (ACE) and Chao 1 (species richness estimator) number of OTUs (Table 2). Regarding bacterial community, the blank group showed higher OTUs than that of the H group in the early periods (about 0 to 25 days) at species level; however, at the end of the digestion, the two groups exhibited similar OTUs. The high number of OTUs, ACE and Chao 1 reveals that, without the supplement of ammonium acetate, no inhibition effect occurred and the abundance of diversity was high in the early periods. While the VFAs accumulated during the AD, the abundance decreased, as reflected by a reduced number of OTUs, ACE and Chao 1. Contrary to the blank group, the H group was supplied with ammonium acetate at the beginning (10 days), and this supplement exhibited strong inhibition on the abundance (low number of OTUs, ACE and Chao 1 in Table 2). Although the VFAs accumulated during the AD, the increased concentration of ammonium formed a buffer system with VFAs, which facilitate growth of certain species in the late phase (50 days), and led to an increase of abundance of diversity (a high number of OTUs, ACE and Chao 1). In addition, the blank group maintained a higher Shannon index compared with the H group during the whole process, which also indicates that the ammonium acetate supplement caused a simpler community structure than that without ammonium acetate supplement.

**Table 2:** **Effective DNA sequences, OTUs (Operational taxonomic units), ACE (abundance-based coverage estimator), Chao 1 (species richness estimator), Shannon (diversity index) and GOOD’s coverage (percent of total species represented in the samples) of samples**

**Sample code**		**Effective reads**	**2,700 effective reads/sample (3% cut-off)**				
			**OTUs**	**ACE**	**Chao 1**	**Shannon**	**GOOD’s coverage**
Blank group	10 days	3,975	794	3,239.3	1,964.1	5.49	81%
	25 days	5,392	780	2,898.1	1,722.2	5.61	82%
	50 days	3,469	611	1,488.0	1,152.0	5.43	88%
H group	10 days	2,941	461	902.7	722.4	5.01	92%
	25 days	3,074	499	1,247.8	878.5	4.83	91%
	50 days	4,852	617	1,990.8	1,209.8	5.15	87%

Figure 3 illustrates the relative taxonomic abundances of the bacterial community at the phylum level. *Phyla Firmicutes*, WWE1, *Bacteroidetes*, *Proteobacteria*, and *Spirochetes* (with relative abundance of higher than 10% in one sample) dominated in both the blank and H groups, and these five phyla are the common bacteria in AD [5,6]. However, ammonium acetate as supplement caused a change of phyla abundance. Although the alike responses of the low biogas production in both blank and H groups were observed, the inhibition could be attributed to different factors.

In the case of the blank group, the *Firmicutes phylum* was the dominant bacteria during the whole process, with a relative abundance of about 44 to 59%, while the relative abundance of *Bacteroidetes* at about 13 to 14% was the second largest phylum. The reason for this was probably that the lack of N-resource resulted in the overgrowth of *Firmicutes* in AD. However, the relative abundance of several dominant phyla in the H group did not show a significant difference (Figure 3); the most dominant bacteria were *Proteobacteria*, whose relative abundance was only about 16 to 31%. The ammonium acetate supplement influenced the growth of particular microbes, which resulted in the different patterns of phyla distributions between the blank and H groups. Furthermore, the gradual changes of the bacterial community during the process is worthy of attention.

**Figure 4:**
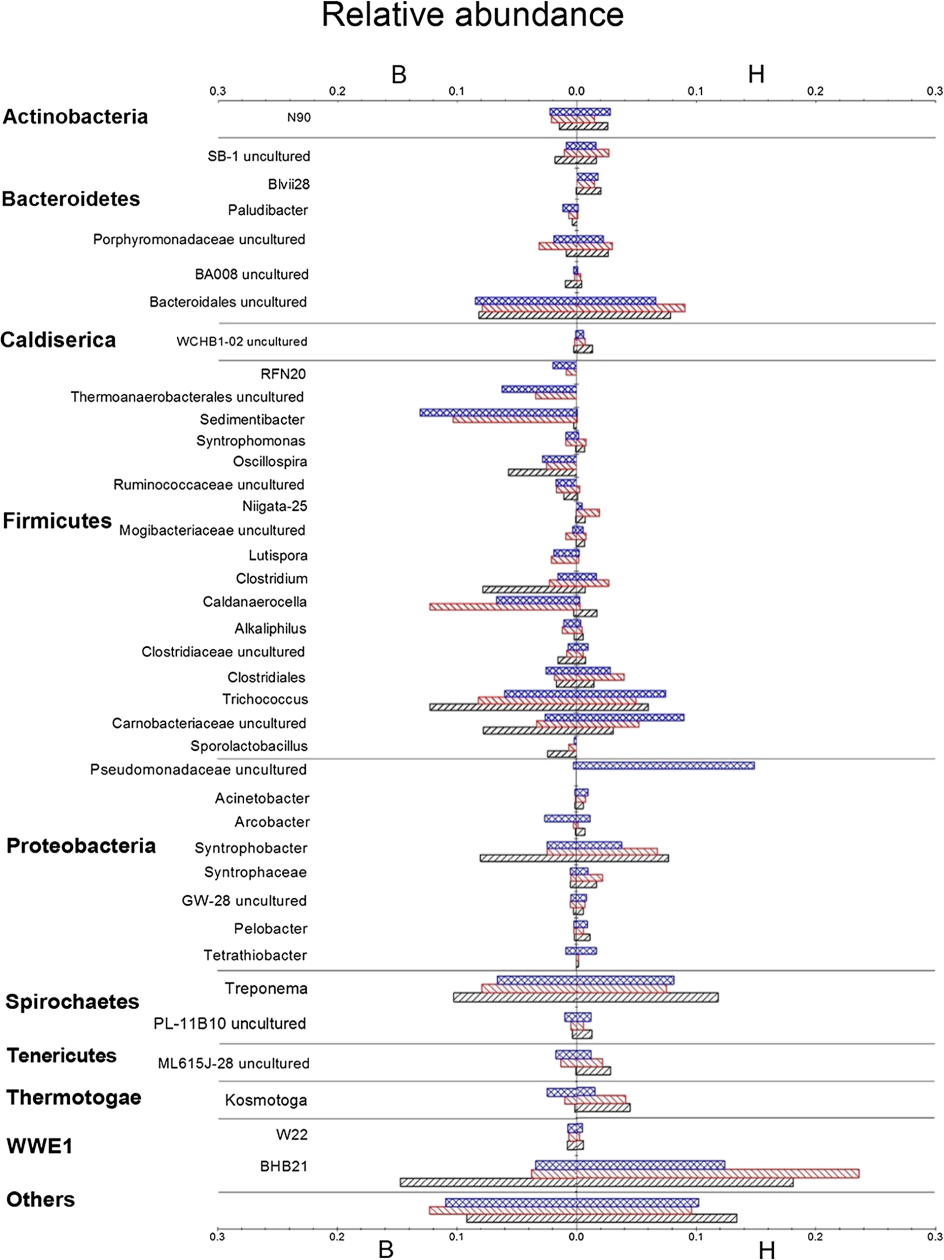
**Taxonomic composition of bacterial community at the genus level from the sequencing.** Taxonomic compositions of bacterial community at the genus level from the sequencing, the left side of the axis shows the results of blank group, the right side of the axis shows the results of H group (brown rectangle symbol: 10 days; red rectangle symbol: 25 days; blue rectangle symbol: 50 days). The length of each bar indicates the relative abundance.

Figure 4 illustrates more details about the change of the blank and H groups at genus level. For the blank group, from 10 to 25 days, *Firmicutes* (as the most abundant phylum) increased from 44 to 59%, which could be attributed to the significant increase of the proportion of *Caldanaerocella* and *Sedimentibacter* from 0.2 to 12.3% and 0.2 to 10.3%, respectively. As a fermentative bacteria, *Sedimentibacter* sp. consumes pyruvate and amino acids for acetate and butyrate production [21]. The relative abundance of *Proteobacteria* and WWE1 decreased from 10 to 25 days, because *Syntrophobacter* and BHB21 decreased from 8.0 to 2.4% and 14.8 to 3.8%, respectively. During this period of AD, the inhibition on methane production of the blank group was not obvious. However, the acid-generating species such as *Sedimentibacter* sp., *Clostridium* sp. reduced the pH value in the blank group. As a consequence, the acidic environment further affected the bacterial community, leading to an increase of the adaptive species and a decrease of the non-adaptive species.

From 25 to 50 days, the community in the blank group changed slightly compared with the early period. The abundance of *Caldanaerocella* decreased from 12.3 to 6.7%, while the abundance of *Sedimentibacter* increased from 10.3 to 13.1%. In the same period, however, the daily average biogas and methane production met a severe decrease, and fluctuation of pH was also observed. This illustrated that the inhibition of biogas production was not as sensitive as the microorganisms’ change to the operating conditions from 25 to 50 days.

The abundance of each species in the H group also shifted during the whole operating process, although a far greater evenness of species was observed in the sample of the H group (Figure 4). The change of bacterial community in the H group was not as significant as that in the blank group from 10 to 25 days. At species level, the abundance of *Treponema* decreased from 11.9 to 7.5%, while BHB21 increased from 18.2 to 23.7%. This indicated that the microbial communities in the H group were relatively stable at the beginning of AD. As described above, according to the experiment data of biogas production and liquid characteristics, the H group was not inhibited in this period, in which biogas production was similar to L and M. This reveals that an appropriate ammonium acetate concentration maintained the balance of microbial ecology.

Compared with 25 days, the microbial communities’ obvious change at 50 days was shown from Table 3. *Pseudomonadaceae* uncultured bacterium was not detected at 25 days, however, its abundance reached 14.9% in 50 days. In addition, BHB21 decreased from 23.7 to 12.4%. The biogas production of the H group was lower than that of the L and M groups since the buffer system was broken down during the same period. However, the change of bacterial population lagged behind the shift of AD characters. On the other hand, along with increased NH_4_^+^ concentration, it was shown that the ecological balance was gradually destroyed from the excess ammonium acetate supplement.

**Table 3:** **Taxonomic composition of the archaeal community at the genus level for the sequences**

	**Relative abundance of H group**		
**Genus**	**10 days**	**25 days**	**50 days**
*Methanosaeta*	82.19%	82.52%	63.56%
*Methanoculleus*	9.85%	10.30%	28.41%
*Methanolinea*	4.04%	3.15%	3.15%
*Methanomicrobiales* uncultured	2.22%	1.96%	3.44%
Coverage	98.30%	97.93%	98.56%
Other	1.70%	2.07%	1.44%

Although the blank and H groups were inhibited and resulted in a similar poor biogas production, there was an essential distinction regarding to the microbial community structure. In the blank group, along with the VFAs accumulation, the microbial communities changed immediately, and thereafter the biogas production was inhibited. However, the system maintained a relatively constant microbial community in the inhibitory period. On the contrary, in the H group, the buffer formed from the supplement offset the impact of the VFAs and stabilized the balance of the microbial colony. Further, the microbial communities in the H group were sensitive when the NH_4_^+^ concentration increased, and drastic species changes were detected when the H group was inhibited, which means that the bacterial community in the blank and H groups was different because of ammonium acetate supplement, especially in the similar VFAs concentration range within 20 days.

The microbial community structure was not the unique factor for the biogas production. The pyrosequencing analysis indicated that an appropriate ammonium acetate concentration not only optimized the community structure, but also promoted the AD activity. Therefore, finely controlled ammonium acetate supplement will facilitate biogas production.

### Archaeal *16S rRNA* gene analysis by pyrosequencing

As described above, when the NH_4_^+^ concentration was beyond the tolerance of the system, the balance of the bacterial community was broken with the change of community structure. According to the low biogas production in the H group, the reason for this may be that the formation and the breakdown of the buffer system also resulted in the change of the aerogenes community, especially methanogen. In the following section, the archaeal community structure was investigated in order to find the relationship of the supplement strategies and the change of the archaeal community.

The relative taxonomic abundances of the archaeal community at genus level were showed in Table 3. It was clear that, the predominant archea included four genera (>1%) before 10 days, *Methanosaeta, Methanoculleus, Methanolinea* and uncultured Archaea of the order *Methanomicrobiales*, all of which were in the phylum *Euryarchaeota*. The relative abundance of *Methanosaeta,* which only consumes acetate for methane production [22] and shows a much lower minimum threshold for acetate utilization, reached up to 82.2% [23]. As reported [8,24], because of the comparison with other aceticlastic methanoarchaea, *Methanosarcina* showed a lower metabolic activity in the high acetate concentrations condition [25]. In this study, seed sludge had been used for the food waste treatment for over one year, and the archaeal community had acclimated at a low acetate concentration during the operation process, which caused *Methanosaeta* to dominate in the community at the beginning of the digestion, before 25 days. At this period, the buffer system formed by ammonium acetate maintained a constant archaeal community in the reactor.

However, when the system was blocked at about 50 days, the proportion of *Methanosaeta* decreased from 82.5 to 63.6%, and *Methanoculleus* increased from 10.3 to 28.4%. It indicated that, with the release of free VFAs, the archaeal community shifted from acetoclastic methanogens to hydrogenotrophic methanogens, as in the research of Kim *et al*. [26], and the free NH_4_^+^ also enhanced the inhibition of acetoclastic methanogens [27]. However, the formation of the buffer delayed the change of archaeal community. When the NH_4_^+^ concentration reached 1.7 g/L, the hydrogen-consuming methanoarchaea maintained more activity than the aceticlastic methanoarchaea [28]. With the breakdown of the buffer (NH_4_^+^/VFAs), the abundance of *Methanosaeta* decreased and *Methanoculleus* became a significant genus, which means that the appropriate ammonia acetate supplement protected both bacteria and archaeal communities. However, excessive addition affected the community of bacteria and archaea negatively, and decreased both biogas and methane production.

## Conclusions

The research of ammonium acetate acting as an efficient supplement demonstrated that the characteristics of a semi-continuous AD operation were significantly influenced. The results showed that an ammonium acetate concentration of 0.7 g/L/d could maintain a stable digestion process, with an average daily methane production of 1,122 mL/d; 44.5% higher than that of the blank group. The present study indicated that the optimal ammonium acetate concentration was about 2.5 to 3.0 g/L. The absence or overload of NH_4_^+^/VFAs led to the inhibition or failure of the AD operation. The DGGE and pyrosequencing analyses of bacterial and archaeal communities indicated the different inhibition in the blank and H groups. The ammonium acetate supplement could cause a simpler community structure than that without ammonium acetate supplement, finally resulting in the different patterns of phyla distributions between the blank and the H groups. The results demonstrate that an appropriate ammonium acetate supplement strategy can be applied to adjust the AD operation and microbial ecology towards the optimal biogas production.

## Methods

### The anaerobic digestion of food waste

The seed sludge used in this study was collected from the Shunyi wastewater treatment plant in Beijing, China, and had been anaerobically adapted to food waste treatment over one year in the laboratory. The characteristics of the seed sludge were assessed in Wang *et al*. [17] and are listed in Table 4. Seed sludge was separated into eight reactors (Shengda Glassware Factory, Shandong province, China) with a working volume of 1 L, and total solids (TS) of 19 wt%, operated at a mesospheric temperature of 37 ± 1°C for the semi-continuous AD. Four groups were examined in duplicate: the blank group with only a glucose feed, and three test groups, where glucose was supplemented by ammonium acetate (Xilong Chemical Co., Ltd, Guangdong province, China) in increasing concentrations of 0.7 g/L/d (L group), 1.0 g/L/d (M group) and 1.3 g/L/d (H group). The feedstock was diluted by the supernatant of the reactor, and then added to the reactor semi-continuously every 24 hours. All systems maintained the same total organic carbon (TOC) load (1.2 g TOC/d) with different N-resource concentrations. The reactors were rubber-sealed. Two sampling tubes were installed: one tube connected to a gas bag for biogas collection, and the other tube (under the liquid surface) for the collection of liquid samples. Gas samples were analyzed every 24 hours, while liquid samples were analyzed every 72 hours.

**Table 4:** **Characteristics of seed sludge** [17] **(TS, Total solids; VS, volatile solids; VFAs, Volatile fatty acids)**

**Parameter**	**Value**
TS (% wet weight)	19
VS (% wet weight)	17
pH	7.3
NH_4_^+^ (g/L)	0.8
VFAs (g/L)	0

### Analytical methods

The TS and volatile solids (VS) content were measured according to Standard Method 2540 G (Shanghai Jing Hong Laboratory Instrument Co., Lid, Shanghai, China) [17]. The pH was measured using a pH probe (MP5C1, Shanghai San-Xin Instrumentation, Inc, Shanghai, China). The NH_4_^+^ was measured according to the Nessler’s reagent colorimetric method of the China State Environmental Protection Administration (GB7479-87). The VFAs and the composition of biogas were measured by gas chromatograph (GC-2014C, SHIMADZU, Kyoto, Japan) [17].

### Microbial structure analyses by denatured gradient gel electrophoresis and pyrosequencing

The extraction of total genomic DNA was carried out by Soil DNA extraction kit (OMEGA, Georgia, United States). For DGGE analysis, the extracted DNA was used for PCR with the primers 341 F-GC/518R (Table 5). To ensure the PCR amplification for archaeal DGGE analysis, a nest PCR was carried out using the primers ARCH46F/ARCH1017R in the first round, and using the primers PARCH344F-GC/UNIV 522R in the second round. The PCR products were analyzed using Bio-Rad Dcode TM Universal Mutation Detection System (Bio-Rad, California, United States). Two polyacrylamide (Amresco, Ohio, United States) gel solutions with concentration of 8% were prepared, and urea (Amresco, Ohio, United States) and deionized formamide (Amresco, Ohio, United States) (containing 7 mol/L urea and 40% formamide, defined as the denaturant concentration 100%) were added in one of the two solutions. The gel was prepared by using the Bio-Rad gradient mixing device (Bio-Rad, California, United States), to make the bacterial denaturant concentration range of approximately 40 to 65%, wherein the concentration of denaturant was decreasing from bottom to top. The sheet was put into a completely solidified electrophoresis tank containing 1 × Tris-Acetate-EDTA buffer (Biotopped, Beijing, China), and the temperature was maintained at 60°C during the whole electrophoresis. Pre-run was performed for 20 minutes under conditions of 180 V, in order to remove impurities in the gel. The 30 μL samples were loaded to the inlet. After electrophoresis at 180 V for 5.5 hours, the gel was stained for 30 minutes using a 3 × GelRed (Biotium, California, United States) for further analysis.

**Table 5:** **The primer sequences for PCR analysis**

**Primer**	**Sequence (5’-3’)**
341 F-GC	CCTACGGGAGGCAGCAG
518R	ATTACCGCGGCTGCTGGC
ARCH46F	YTAAGCCATGCRAGT
ARCH1017R	GGCCATGCACCWCCTCT
PARCH344F-GC	CGCCCGCCGCGCGCGGCGGGCGGGGCG
	GGGGCACGGGGGGHGCAGCAGGCGCGA

For pyrosequencing, the DNA extracted by the above mentioned method was used for amplification of the bacterial hypervariable region with the primers 341 F-GC/518R and the archaeal hypervariable region with the primers ARC344/ARC915 of the *16S rRNA* gene (Table 5). Specific 10-bases long barcode sequences were attached to primers as a tag for identification. The PCR products were sent to Hanyu biotech Co. Ltd. (Shanghai, China) for pyrosequencing using a 454 GS-FLX sequencer (Roche Diagnostics Co., Indiana, United States) using a Titanium Sequencing Kit (Roche Diagnostics Co., Indiana, United States). The method for use of the DNA extraction kit, and the comparison by extraction of DNA from archaeal sludge, was illustrated previously [29].

In the obtained pyrosequencing data, ambiguous and short sequences with a length less than 200 and quality score of less than 20 nucleotides were removed. In addition, qualified sequences were clustered into OTUs defined by a 3% distance level using complete-linkage clustering. Distance matrices were generated and sequences were grouped to generate rarefaction curves by a 3% distance level [30]. OTU-based diversity analyses and richness estimates of Chao 1 and ACE, the Shannon index and Simpson index calculations were performed using the mothur package (www.mothur.org/wiki/Download_mothur) [31].

## Abbreviations

ACE: Abundance-based coverage estimator
AD: Anaerobic digestion
DGGE: Denatured gradient gel electrophoresis
OTUs: Operational taxonomic units
PCR: Polymerase chain reaction
TAE buffer: Tris-Acetate-EDTA buffer
TOC: Total organic carbon
TS: Total solids
VFAs: Volatile fatty acids
VS: Volatile solids
